# Human Papillomavirus Vaccination Before and During the COVID-19 Pandemic

**DOI:** 10.1001/jamanetworkopen.2022.34000

**Published:** 2022-09-19

**Authors:** Jenny K. R. Francis, Sitara M. Weerakoon, Serena L. Lucas, Matthew S. Mathew, Julia C. Durante, Nancy Kelly, Jasmin A. Tiro

**Affiliations:** 1 Department of Pediatrics, University of Texas Southwestern Medical Center, Dallas; 2 Children’s Health System of Texas, Dallas; 3 University of Texas Health Science Center School of Public Health, Dallas; 4 Center for Pediatric Population Health, Dallas, Texas; 5 Department of Population and Data Sciences, University of Texas Southwestern Medical Center, Dallas

## Abstract

This cross-sectional study compares human papillomavirus vaccinations by age and season before and during the COVID-19 pandemic.

## Introduction

Every clinical encounter is an opportunity to vaccinate. The COVID-19 pandemic disrupted in-person encounters, leading to delays in childhood vaccinations across different seasonal patterns.^1,2^ Historically, human papillomavirus (HPV) vaccination has lagged behind other adolescent vaccinations.^3^ Strategies to catch up by offering vaccines at every encounter starting at age 9 years^4,5^ and prioritizing population subgroups are needed. We characterized HPV vaccination by age and season from 2019 to 2021 and compared vaccination by encounter before and during the COVID-19 pandemic to identify catch-up priority groups.

## Methods

This cross-sectional study assessed encounters from January 2019 to December 2021 of patients aged 9 to 22 years at Children’s Health Medical Group, Dallas, Texas, who were seen for well or follow-up visits, with no change in HPV vaccine supply or availability during the study period. The study was approved by the University of Texas Southwestern Medical Center institutional review board, with a waiver of informed consent because this was a nonregulated quality improvement project. The STROBE reporting guideline was followed.

Structured queries of electronic health records were used to identify patients due for an HPV dose and whether it was received at an encounter. Demographic characteristics and encounter features (eg, order during study period) associated with HPV vaccination were assessed using Mann-Whitney tests adjusted for nonindependence of observations. To understand pandemic effects and seasonal variation, difference-in-difference testing compared total vaccines administered each season in each year.^2^ A priori significance was set at 2-sided *P* ≤ .05. Analyses were performed used Stata, version 15.

## Results

Among 4548 patients with 10 469 encounters (Table 1), the percentage receiving HPV vaccination was higher in 2021 (1118 of 3522 [35.0%]) and 2020 (1182 of 3320 [35.6%]) compared with 2019 (1222 of 3957 [30.9%]) (*P* < .001) despite a 19.3% decrease in the number of encounters in 2021 (n = 3192) compared with 2019 (n = 3957). The youngest eligible age group (9-10 years) had the lowest percentage of vaccinations compared with other age groups, representing 3629 of 10 469 encounters (34.7%) but only 10 of 3522 vaccinations (0.3%).

**Table 1.** Demographic Characteristics of Patients Aged 9 to 22 Years Who Had Eligible Encounters for an HPV Vaccine Dose by Vaccination Status at the End of the Encounter^a^

Characteristic	Encounters, No. (%)		*P* value^c^
	HPV vaccine dose not received (n = 6947)	HPV vaccine dose received (n = 3522)^b^	
Age, y^d^			
9-10	3619 (52.1)	10 (0.3)	<.001
11-12	1833 (26.4)	2190 (62.2)	
13-14	645 (9.3)	834 (23.7)	
15-17	589 (8.5)	406 (11.5)	
≥18	261 (3.8)	82 (2.3)	
Sex			
Female	3180 (45.8)	1675 (47.6)	.09
Male	3767 (54.2)	1847 (52.4)	
Race and ethnicity^e^			
Hispanic			.01
White	2835 (40.8)	1539 (43.7)	
Other^f^	557 (8.0)	288 (8.2)	
Non-Hispanic			
Black	2410 (34.7)	1180 (33.5)	
White	436 (6.3)	195 (5.5)	
Other^g^	656 (9.4)	301 (8.5)	
Insurance at first encounter			
Commercial	1553 (22.4)	793 (22.5)	.62
Government	5322 (76.6)	2705 (76.8)	
Other	72 (1.0)	24 (0.7)	
Encounter year			
2019	2735 (39.4)	1222 (34.7)	<.001
2020	2138 (30.8)	1182 (33.6)	
2021	2074 (29.9)	1118 (31.7)	
Encounter season^h^			
Winter	1196 (17.2)	609 (17.3)	<.001
Spring	1254 (18.1)	679 (19.3)	
Summer	1803 (26.0)	1098 (31.2)	
Fall	2694 (38.8)	1136 (32.3)	
Order of encounter			
First	3148 (45.3)	1400 (39.8)	<.001
Second to fifth	3525 (50.7)	1948 (55.3)	
Sixth to 15th	274 (3.9)	174 (4.9)	

Abbreviation: HPV, human papillomavirus.

^a^ Eligible encounters represent well child or follow-up visits. Data are for 4548 unique patients with encounters from January 1, 2019, to December 31, 2021.

^b^ Some patients were counted twice (first dose given at 1 encounter and second dose given at next encounter).

^c^ Mann-Whitney nonindependence test.

^d^ Age at the end of each encounter year (December 31). Patients aged 9 to 10 years were eligible for vaccination; 11 to 12 years, on time for vaccination; 13 to 14 years, last opportunity for 2-dose series; and 15 to 17 years, eligible for 3-dose series.

^e^ Race and ethnicity data were missing for 125 encounters (72 unique patients). Race and ethnicity were ascertained by self-report and were included in the analysis to evaluate historical disparities between race, ethnicity, and vaccinations.

^f^ Preferred not to answer or unknown.

^g^ Included Asian, American Indian and Alaska Native, preferred not to answer, unknown, or other.

^h^ Winter was from January 1 to March 11; spring, March 12 to May 31; summer, June 1 to August 31; and fall, September 1 to December 31.

Table 2 compares the adjusted percentage change in vaccinations per season. In 2020 compared with 2019, there was an 17.99% increase in vaccinations during the winter. At the beginning of the COVID-19 pandemic in spring and summer 2020, vaccinations changed by −37.31% and −22.30%, respectively, and by 37.37 % in fall. In 2021 compared with 2020, vaccinations changed by −11.66 % in winter, 44.64% in spring, −3.85% in summer, and −28.33% in fall. In 2021 compared with 2019 (before the pandemic), vaccinations changed by 4.23% in winter, −9.33% in spring, −25.29% in summer, and 6.97% in fall.

**Table 2.** Comparison of Encounters in Which an HPV Vaccine Dose Was Administered to a Patient at a Pediatric Primary Care Clinic by Encounter Year and Season^a^

	Encounters, No.			Difference, % (rate ratio)^b^		
	2019	2020	2021	2020 vs 2019	2021 vs 2020	2021 vs 2019
**Winter**						
Total sample	189	223	197	17.99 (1.18)	−11.66 (0.88)	4.23 (1.04)
Age, y^c^						
9-10	0	0	1	0 (1.00)	100.00 (2.00)	100.00 (2.00)
11-12	95	97	99	2.10 (1.02)	2.06 (1.02)	4.21 (1.04)
13-14	70	77	58	10.00 (1.10)	−24.68 (0.75)	−17.14 (0.83)
15-17	19	39	32	105.26 (2.05)	−17.95 (0.82)	68.42 (1.68)
**Spring**						
Total sample	268	168	243	−37.31 (0.63)	44.64 (1.45)	−9.33 (0.91)
Age, y^c^						
9-10	1	1	2	0 (1.00)	100.00 (2.00)	100.00 (2.00)
11-12	154	104	151	−32.47 (0.68)	45.19 (1.45)	−1.95 (0.98)
13-14	66	42	57	−36.36 (0.63)	35.71 (1.36)	−13.63 (0.86)
15-17	38	13	29	−65.79 (0.34)	123.08 (2.23)	−23.68 (0.76)
**Summer**						
Total sample	435	338	325	−22.30 (0.77)	−3.85 (0.96)	−25.29 (0.75)
Age, y^c^						
9-10	1	1	0	0 (1.00)	−100.00 (0.00)	−100.00 (0.00)
11-12	272	228	218	−16.18 (0.84)	−4.39 (0.95)	−19.85 (0.80)
13-14	96	72	74	−25.00 (0.75)	2.78 (1.03)	−22.92 (0.77)
15-17	49	31	31	−36.73 (0.63)	0 (1.00)	−36.73 (0.63)
**Fall**						
Total sample	330	453	353	37.37 (1.37)	−28.33 (0.78)	6.97 (1.07)
Age, y^c^						
9-10	1	2	1	100.00 (2.00)	−50.00 (0.50)	0.00 (1.00)
11-12	222	303	247	36.49 (1.36)	−18.48 (0.82)	11.26 (1.11)
13-14	64	94	64	46.88 (1.47)	−31.91 (0.68)	0.00 (1.00)
15-17	39	49	37	25.64 (1.26)	−24.49 (0.76)	−5.13 (0.94)

Abbreviation: HPV, human papillomavirus.

^a^ Data are from January 1, 2019, to December 31, 2021. Periods match the seasons of the 2020 pandemic year: winter, prepandemic (January 1 to March 11); spring, stay-at-home orders (March 12 to May 31); summer, reopening (June 1 to August 31); and fall, return to school (September 1 to December 31).

^b^ Percentage difference was calculated as ([(*n*2 − *n*1) / *n*1] * 100), and rate ratio was calculated as *n*2/*n*1, where *n*1 indicates the number of encounters in the earlier year and *n*2 represents the number of encounters during the later year.

^c^ Age was classified at the end (December 31) of each encounter year. Patients aged 9 to 10 years were eligible for vaccination; 11 to 12 years, on time for vaccination; 13 to 14 years, last opportunity for 2-dose series; and 15 to 17 years, last opportunity for 3-dose series. Data for patients aged 18 years or older are not reported owing to small sample size, but the total sample includes this age group.

## Discussion

We found a steady increase in HPV vaccinations per encounter between 2019 and 2021 despite an 19.3% decrease in overall encounters. The pandemic may have been associated with providers feeling pressured to not miss vaccination at in-person encounters. The number of unique patients was unchanged during the pandemic, which was likely true for other settings. A study limitation is that encounter-level data are useful for clinicians to understand day-to-day vaccine performance but cannot be compared with patient-level state or national data.

Patients aged 9 to 10 years were the least vaccinated even though the practice recommended vaccinations at all encounters. Future studies with larger samples should evaluate age and sex together to assess disparities. Also, vaccination levels in summer of 2020 and 2021 did not catch up to prepandemic vaccination levels in 2019 despite summer typically having more well visits and sports-related examinations. Of note, HPV vaccinations in winter 2020 vs 2019 were largely unchanged, a control for this unadjusted analysis. Efforts to catch up and surpass prepandemic vaccination levels should consider age and seasonality to forecast patient need and adjust vaccine stock and storage to ensure HPV vaccination does not decrease further.

## References

[1] Nelson R. COVID-19 disrupts vaccine delivery. Lancet Infect Dis. 2020;20(5):546. doi:10.1016/S1473-3099(20)30304-2 32311326 PMC7164887

[2] Ackerson BK, Sy LS, Glenn SC, . Pediatric vaccination during the COVID-19 pandemic. Pediatrics. 2021;148(1):e2020047092. doi:10.1542/peds.2020-047092 33858983

[3] Pingali C, Yankey D, Elam-Evans LD, . National, regional, state, and selected local area vaccination coverage among adolescents aged 13-17 years—United States, 2020. MMWR Morb Mortal Wkly Rep. 2021;70(35):1183-1190. doi:10.15585/mmwr.mm7035a1 34473682 PMC8422873

[4] O’Leary ST, Nyquist AC. Why AAP recommends initiating HPV vaccination as early as age 9. AAP News. Accessed May 18, 2022. https://publications-aap-org.foyer.swmed.edu/aapnews/news/14942

[5] American Cancer Society. HPV vaccination at 9-12 years of age. Accessed May 18, 2022. https://hpvroundtable.org/wp-content/uploads/2022/04/Evidence-Summary-HPV-Vaccination-Age-9-12-Final.pdf

